# *Mycoplasma genitalium* and *Chlamydia trachomatis* infection among women in Southwest China: a retrospective study

**DOI:** 10.1017/S0950268822001066

**Published:** 2022-06-23

**Authors:** Guanglu Che, Fang Liu, Qiuxia Yang, Shuyu Lai, Jie Teng, Yuan Tan, Jiaxin Duan, Li Chang

**Affiliations:** Department of Laboratory Medicine, West China Second University Hospital, Key Laboratory of Birth Defects and Related Diseases of Women and Children, Ministry of Education, West China Second University Hospital, Sichuan University, Chengdu, Sichuan 610041, P.R. China

**Keywords:** *Chlamydia trachomatis*, high-risk human papillomavirus, infection, *Mycoplasma genitalium*, vaginal secretions

## Abstract

*Mycoplasma genitalium* (MG) and *Chlamydia trachomatis* (CT) are the most common sexually transmitted pathogens, which can cause cervicitis, pelvic inflammation and infertility in female. In the present study, we collected the basic information, clinical results of leucorrhoea and human papillomavirus (HPV) infection of patients, who were involved in both MG and CT RNA detection in West China Second Hospital of Sichuan University from January 2019 to April 2021, ranging from 18 to 50 years old. The results showed that the infection frequencies of MG and CT were 2.6% and 6.5%, respectively. The infection rate of CT in gynaecological patients was significantly higher than that of MG (*P* < 0.001). Moreover, patients with CT infection often had symptoms of gynaecological diseases, while patients with MG infection remain often asymptomatic. By exploring the connection between MG or CT infection and vaginal secretions, we found that the infection of MG or CT promoted to the increase of vaginal leukocytes, and CT infection exacerbated the decrease of the number of *Lactobacillus* in the vagina. Further analysis suggested that independent infection and co-infection of MG or CT resulted in abnormal vaginal secretion, affecting the stability of vaginal environment, which may induce vaginal diseases. Unexpectedly, our study found no association between MG or CT infection and high-risk HPV infection. In conclusion, our study explored the infection of MG and CT among women in Southwest China for the first time, and revealed that the infection of MG or CT would affect the homeostasis of vaginal environment, which laid a foundation for the clinical diagnosis and treatment of MG and CT infection.

## Introduction

Sexually transmitted diseases (STD) are gradually increasing worldwide, which mainly cause urogenital infections in women and men. More and more pathogens are involved in the pathogenesis of STD, such as mycoplasma, chlamydia, bacteria, fungi and spirochetes. Especially *Mycoplasma genitalium* (MG) and *Chlamydia trachomatis* (CT) are not only considered as fundamental sexually transmitted pathogens causing non-gonococcal urethritis (NGU) in men, but also associated with cervicitis, pelvic inflammatory disease and infertility in women.

MG is the smallest prokaryote and the smallest mycoplasma in the genome and first isolated from men with NGU in 1981. Because MG, which needs special media and long incubation time, is difficult to culture, the diagnosis of MG infection depends on nucleic acid amplification. A growing number of studies showed that MG is closely related to female reproductive tract infection, female infertility and pregnancy outcome [1–4]. At present, the patients in medical institutions are mainly included in the population and objects of epidemiological research, rather than the general population. The prevalence of MG infection in women from different groups ranged from 1% to 16% according to epidemiological survey results [5–7]. However, there is a lack of epidemiological data on the prevalence of MG among women in Southwest China.

CT infections are the most common sexually transmitted bacterial infections worldwide, which cause urogenital diseases, such as NGU, vaginitis, cervicitis and endometritis. Some studies demonstrated that CT not only leads to premature rupture of membranes, premature delivery and neonatal death, but also caused neonatal conjunctivitis and neonatal pneumonia by mother to child transmission. According to the epidemiological survey of CT infections, the prevalence of CT infection varies from 3% to 35% in women from different countries [8–11].

More and more literature has reported that genital tract MG infection is often accompanied by co-infection of other pathogens, among which CT is one of the important co-infection pathogens [12–15]. At present, the research on the co-infection of MG and CT in genital tract only focuses on the investigation of co-infection rate and drug resistance. However, studies on the association between MG or CT infection and vaginal cleanliness are insufficient. In the present study, the general information, leucorrhoea examination data and high-risk human papillomavirus (HPV) infection of women diagnosed as MG or CT infection by simultaneous amplification and testing in West China Second Hospital of Sichuan University from January 2019 to April 2021 were collected; and the infection rate of MG and CT, the age distribution of MG and CT infection, the correlation with vaginal cleanliness and high-risk HPV infection were retrospectively analysed.

## Methods and materials

### Study subjects and data collection

The study subjects were involved in both MG and CT RNA detection in West China Second Hospital of Sichuan University, the largest gynaecological diagnosis and treatment centre in Southwest China, from January 2019 to April 2021, ranging from 18 to 50 years old. Exclusion criteria: (1) pregnancy; (2) the age is less than 18 years old or more than 50 years old; (3) only MG or CT RNA was detected; (4) duplicate detection. Our study was approved by Medical Ethics Committee of West China Second University Hospital, Sichuan University.

The data were collected from each subject, including age, basic clinical information, vaginal cleanliness of leucorrhoea examination, the number of vaginal leukocytes, vaginal Lactobacilli and vaginal clue cells and high-risk HPV infection.

### MG and CT RNA detection

(1) Specimen collection: Insert a medical cotton swab 1–2 cm into the endocervical canal, gently rotate the swab once and hold it for 10 s, and then withdraw the swab carefully, store it into 2 ml of special sample preservation solution. (2) The sample processing and RNA extraction were carried out according to the instructions of isothermal RNA amplification assay for MG and CT commercial test kits (Rendu Biotechnology, Shanghai, China). The kit was approved by China food and drug administration. (3) Amplification and detection: The 16sRNA of MG or CT is considered to be the target genes for amplification. Set the reaction program as 40 cycles of incubation at 42 °C for 1 min each cycle. Select the FAM channel for fluorescein, and fluorescence signals are to be recorded once per minute. When Ct values are ⩽35, MG RNA or CT RNA is considered positive. When Ct values are 35<*Ct*<40, samples need to be retested. It is considered positive if *Ct*<40 in retesting.

### Leucorrhoea examination

Specimen collection: Vaginal secretion was scraped from the upper third of the vaginal wall with a sampling swab and placed in the sampling tube for immediate examination. (2) Preparation of vaginal smear: The vaginal secretions were smeared immediately. After the smears were dried, they were stained with Gram staining. (3) Microscopic examination: First, *Candida*, *Trichomonas vaginalis*, Gram-negative diplococcus or clue cells were observed with low-power lens (100×). Then the distribution and quantity of leukocytes were observed under high-power lens (400×). The distribution and quantity of *Lactobacillus* and epithelial cells were observed by oil lens (1000×). Finally, the vaginal cleanliness was determined according to the results of microscopic examination. The criteria of vaginal cleanliness are as follows (Table 1), referring to the common vaginal cleanliness judgement modes in the laboratory department of West China Second Hospital of Sichuan University.

**Table 1.** The criteria for vaginal cleanliness

Vaginal cleanliness	Epithelial cells	WBC	*Lactobacillus*	Spores	Budding spores	Pseudohypha	Trichomonas vaginalis	Staphylococcus	Clue cells	Miscellaneous bacterium
I	1/2 or full visual field	0–5/HP	>30/oil lens	None	None	None	None	None	None	None
II	1/2 or full visual field	0–5 or 5–15/HP	6–30/oil lens	None or found	None	None	None	None	None	None or found
III	⩽1/2 visual field	15–30/HP	1–5/oil lens	None or found	None or found	None or found	None or found	None or found	None – >20%	None or found
IV	⩽1/2 visual field	>30/HP	<1/oil lens	None or found	None or found	None or found	None or found	None or found	None – >20%	None or found

HP, high-power lens.

### HC2 for high-risk HPV detection

(1) Specimen collection: Put the cervical brush into the cervical orifice, rotate it three times in the same direction and stay for 10 s. Then the specimens are transported using specimen transport medium. (2) High-risk HPV detection: Nucleic acid denaturation of specimens and detection of high-risk HPV were carried out following the instructions of *digene* HC2 High-Risk HPV DNA Test kit (Qiagen, German) strictly, which can simultaneously detect 13 high-risk HPVs published by world health organisation, including 16, 18, 31, 33, 35, 39, 45, 51, 52, 56, 58, 59 and 68 subtypes. (3) Result interpretation: Specimens with relative light unit/cut-off (RLU/CO) ratios ⩾1.0 are considered positive; specimens with RLU/CO ratios <1.0 are considered negative or non-detected for the 13 high-risk HPV types.

### Statistical analysis

Statistical analysis was performed by applying GraphPad Prism 8.2.1 (GraphPad Software Inc., San Diego, CA, USA). The *χ*^2^ tests were used to determine statistically significant differences in rates or constituent ratios among several groups. The statistical differences of age in three or more groups and two groups were evaluated using non-parametric one-way ANOVA and *Q* test, respectively. *P* < 0.05 was considered to be statistically significant.

## Results

### The basic characteristics of MG or CT infection

**Fig. 1.**
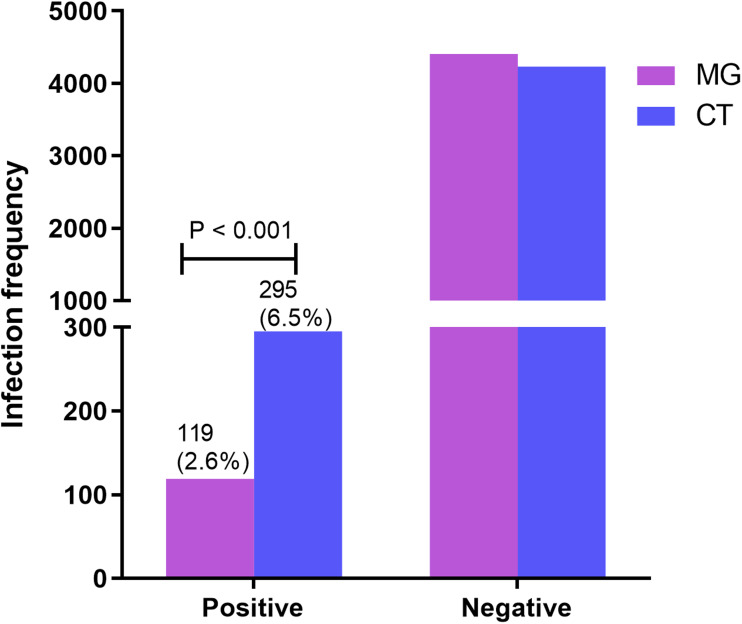
Infection frequencies of MG and CT in gynaecological patients.

A total of 4526 women were tested for MG RNA and CT RNA, collectively. A total of 119 (2.6%) women were detected with MG infection and 295 (6.5%) women were detected with CT infection. Screening results of gynaecological patients showed that the infection rate of CT was significantly higher than that of MG (*P* < 0.001) (Fig. 1). Besides, 92 patients were infected by MG alone, aged 30.5 ± 4.7 years; 268 were independently infected by CT, aged 29.6 ± 5.3 years; and 27 were co-infected by MG and CT, aged 28.3 ± 5.4 years. In total, 4139 people were infected by neither MG nor CT infection, and 500 patients (aged 30.9 ± 5.0 years) were randomly selected to collect clinical data and other relevant examination results for analysis. MG and CT are mainly infected with women aged 25–35 years old with high incidence of sexual behaviour. Although there were significant differences between age and the carrying rate of MG and CT infection in women, little clinical significance was observed between age and carriage rate of MG and CT infection in women (Table 2). We discovered that the majority of women with CT infection (54.9%) sook medical advice because of the symptoms of gynaecological diseases such as vaginitis, cervicitis or pelvic inflammation by disease spectrum analysis. Nevertheless, women with MG infection (35.9%) were more likely to be asymptomatic, compared with women with CT infection (23.5%) (*P* < 0.001, Table 2). Besides, compared with MG or CT infection alone, co-infection of MG and CT can significantly promote female infertility. Surprisingly, there was no significant difference in disease composition between women co-infected with MG and CT and women without MG and CT infection (Table 2).

**Table 2.** The basic characteristics of MG or CT infection

	MG (+) CT (−) (*n* = 92)	MG (−) CT (+) (*n* = 268)	MG (+) CT (+) (*n* = 27)	MG (−) CT (−) (*n* = 500)	*P* value
Mean age^a^	30.5 ± 4.7	29.6 ± 5.3*	28.3 ± 5.4*#	30.9 ± 5.0	<0.01
Disease spectrum^b^					<0.05
Gynaecological diseases	34 (37.0%)	147 (54.9%)*	11 (40.7%)	220 (44.0%)	
Infertility	25 (27.2%)	58 (21.6%)	10 (37.0%)	184 (36.8%)	
Asymptomatic	33 (35.9%)*	63 (23.5%)	6 (22.2%)	96 (19.2%)	

*vs MG (−) CT (−), *P* < 0.05; #: *vs* MG (+) CT (−), *P* < 0.05.

a Means non-parametric one-way ANOVA was used for statistical analysis.

b Means *χ*^2^ test was used for statistical analysis.

### Correlation between MG or CT infection and leucorrhoea examination

Leucorrhoea examination is an important mean to determine whether the leucorrhoea is abnormal, including the number of white blood cells (WBCs) and *Lactobacillus*, clue cell percentage, *Candida*, vaginal cleanliness degree. Microscopic analysis of the number of WBCs in vaginal secretion showed that MG or CT infection can result in a significant increase in WBCs in vaginal secretions, and the number of WBCs promoted by CT significantly exceeded MG. In addition, co-infection of MG and CT can aggravate the increase of WBCs, compared to MG or CT infection alone (*P* < 0.05, Table 3). The results of *Lactobacillus* in vaginal secretion revealed that there was no significant difference in the number of *Lactobacillus* in vaginal secretion between MG single-positive patients and double-negative patients, but the number of *Lactobacillus* in vaginal secretions of CT single-positive patients increased significantly (*P* < 0.05, Table 3). Compared with patients with MG or CT infection alone, patients with MG and CT co-infection had significantly less *Lactobacillus* in leucorrhoea secretion. Besides, we also found that MG or CT was significantly related to the increase of clue cells in vaginal secretion (*P* < 0.001, Table 3).

**Table 3.** Correlation between MG or CT infection and leucorrhoea examination

	MG (+) CT (−) (*n* = 92)	MG (−) CT (+) (*n* = 268)	MG (+) CT (+) (*n* = 27)	MG (−) CT (−) (*n* = 500)	*P* value
WBC^a^					<0.05
⩽15 (<5 or 5–15)	58 (63.0%)	127 (47.4%)	7 (25.9%)	386 (77.2%)	
>15 (15–30 or >30)	34 (37.0%)*	141 (52.6%)*#	20 (74.1%)*#$	114 (22.8%)	
*Lactobacillus*^a^					<0.05
⩾6 (6–30 or >30)	42 (45.7%)	106 (39.6%)	4 (14.8%)	256 (51.2%)	
⩽5 (1–5 or <1)	50 (54.3%)	162 (60.4%)*	23 (85.2%)*#$	244 (48.8%)	
Clue cell^a^	16 (17.4%)*	58 (21.6%)*	7 (25.9%)*	18 (3.6%)	<0.001
Fungal vaginitis^a^	6 (6.5%)	31 (11.6%)*	0	14 (2.8%)	<0.001
Vaginal cleanliness^a^					<0.01
I + II	67 (72.8%)	129 (48.1%)	7 (25.9%)	428 (85.6%)	
III + IV	25 (27.2%)*	139 (51.9%)*#	20 (74.1%)*#$	72 (14.4%)	

*vs MG (−) CT (−), *P* < 0.05; #: *vs* MG (+) CT (−), *P* < 0.05; $: *vs* MG (−) CT (+), *P* < 0.05.

a Means *χ*^2^ test was used for statistical analysis.

Vaginal cleanliness degrees I and II are considered normal leucorrhoea, otherwise degrees III and IV are considered abnormal leucorrhoea. Further analysis by synthesis of vaginal cleanliness found that the patients with vaginal cleanliness degrees I and II were 67 (72.8%), and the vaginal cleanliness of 25 (27.2%) patients was degrees III and IV among the MG single-positive women (Table 3). Out of 268 women with CT infection alone, 129 (48.1%) were detected with vaginal cleanliness of degrees I and II, 139 (51.9%) were detected with vaginal cleanliness of degrees III and IV. The test results of leucorrhoea of 500 women with MG and CT double-negative were also collected. The vaginal cleanliness of 428 (85.6%) women was graded degrees I and II. There were only 72 (14.4%) women with vaginal cleanliness of degrees III and IV. Seriously, among the 27 women co-infected with MG and CT, 20 (74.1%) had vaginal cleanliness of grades III and IV. The results of vaginal cleanliness showed that the infection of MG or CT could lead to the leucorrhoea abnormality. Furthermore, CT infection has a more serious impact on the vaginal cleanliness degree than MG infection and the concomitant infection of MG and CT will more significantly lead to the abnormal vaginal environment (*P* < 0.01; Table 3).

### Correlation between MG or CT infection and high-risk HPV

High-risk HPV is also one of the important pathogens causing reproductive tract infections in women, leading to external genital cancer, cervical cancer and cervical intraepithelial neoplasia. Therefore, we also investigated the relationship between MG or CT infection and high-risk HPV infection. The results demonstrated that there was no significant relationship between MG or CT infection and high-risk HPV infection (*P* = 0.123) (Table 4).

**Table 4.** Correlation between MG or CT and high-risk HPV co-infection

	MG (+) CT (−) (*n* = 92)	MG (−) CT (+) (*n* = 268)	MG (+) CT (+) (*n* = 27)	MG (−) CT (−) (*n* = 500)	*P* value
High-risk HPV^a^					0.123
Negative	73 (79.3%)	218 (81.0%)	20 (74.1%)	422 (84.4%)	
Positive	19 (20.7%)	51 (19.0%)	7 (25.9%)	78 (15.6%)	

a Means χ^2^ test was used for statistical analysis.

## Discussion

In this study, we mainly investigated bacterial sexually transmitted infections (STIs) MG and CT prevalence in women attending our hospital in northwest China. The infection rates of MG and CT were 2.6% and 6.5%, respectively. The prevalence of CT in patients with gynaecological diseases was significantly higher than that of MG. At the same time, there are many reports about the prevalence of MG and CT in women in other countries and regions. The meta-analysis of Baumann *et al*. showed that the overall prevalence of MG in the general population was 1.3% [7]. Moridi *et al*. meta-analysis revealed that the prevalence of MG among Iranian women was 11.3% [16]. The prevalence of MG in pregnant women and female sex workers was 0.9% and 15.9% up to 2017, respectively, according to a systematic review and meta-analysis [7]. Carneiro *et al*. discovered that the positive rate of CT in cervical cytology of samples from women in gynaecological clinic was 10% by multiplex PCR [17]. Besides, it has been reported that the infection rate of CT of young women in STI clinics achieved 18% [18]. Compared with many previous studies, the positive rates of MG and CT in our study were significantly lower. The main explanation is the inconsistency of screening population. In our study, the research object almost consisted of the majority of gynaecological patients, without limitation to patients with reproductive tract infection and infertility. Nevertheless, other studies mainly focused on high-risk groups, such as sex workers, patients with multiple sexual partners or infertility, adverse pregnancy history, etc. The second reason is the divergence caused by different detection methods. Compared with other studies using traditional nucleic acid amplification technology, we detected the RNA of pathogens by performing real-time isothermal amplification technology that can only detect live mycoplasma, but not detect inactivated mycoplasma caused by drug treatment. The limits of the detection (LoD) of the isothermal RNA amplification assay for MG and CT commercial test kits are 1000 copies in our study. Moreover, the study of Hu *et al*. showed that the analytical sensitivity of the multiplex real-time PCR melting curve assay is very splendid, with LoD <200 copies/μl for the DNA of the nine STDs, including MG [19]. Studies have reported that the LoD of CT can reach 50 copies by TaqMan PCR [20]. Compared with PCR method, although the LoD of the isothermal RNA amplification is lower than that of PCR, the isothermal RNA amplification assay only detects the RNA of active pathogens, which is more valuable for the diagnosis of current infection. Besides, the isothermal RNA amplification assay can eliminate the false-positive results caused by the residual DNA of pathogens.

Meanwhile, through the disease spectrum analysis of patients, according to the causes why the patients sook medical advice, we found that CT infection obviously led to the symptoms of gynaecological diseases, while MG infection in women was more likely to be asymptomatic, which is easy to be ignored. The 2016 European guidelines on MG infections also revealed that MG infection was often asymptomatic [21], complicating the diagnosis and treatment of MG infection. However, there was no significant difference in disease spectrum between patients with MG and CT co-infection and patients without MG or CT infection. On the one hand, the classification of disease spectrum was incomplete, in which gynaecological diseases were not limited to vaginitis, cervicitis, pelvic inflammation and so on. On the other hand, the aetiology of gynaecological patients is complex.

At present, there are few studies on the correlation between MG and CT infection and leucorrhoea detection. Our study demonstrated that the infection of MG and CT, which promoted the number of WBCs increase and was also related to the increase of clue cells in vagina, could obviously lead to the abnormality of vaginal secretions. A growing number of studies found that not only the majority of patients with MG infection but also CT infection remain asymptomatic and have no obvious infection symptoms or no disease [18, 22–24]. The treatment of MG and CT infection will be ignored, because of asymptomatic infection, to promote a long-term latent infection, leading to infertility and other adverse consequences. Currently, international guidelines recommend an annual screening for genital CT for high-risk population [25]. The 2016 European guidelines on MG infections clearly pointed out that MG should be regularly screened for high-risk sexual behaviours [21]. Therefore, it is urgent to strengthen the screening of female bacterial STI to protect women's reproductive tract health from MG and CT asymptomatic infection. Our research supported that further screening for MG and CT infection in patients with abnormal vaginal secretions and no symptoms of genital tract has more clinical significance, which is helpful for clinicians to carry out further diagnosis and treatment services. Our results also lay a foundation for further study of the relationship and mechanism between MG or CT infection and vaginal diseases.

HPV is composed of more than 150 different types of small double-stranded DNA viruses, which can cause common STIs and lead to female genital infection, adverse pregnancy outcomes, infertility and other consequences [26–28]. Numerous studies have demonstrated that persistent infection of high-risk HPV can lead to cervical atypical hyperplasia, eventually causing cervical cancer [26, 29, 30]. Meanwhile, an increasing number of studies have found that concomitance CT and HPV infection is a high-risk factor for cervical precancerous lesions [31, 32]. Therefore, we investigated the relationship between high-risk HPV and MG or CT co-infection, establishing the basis for the diagnosis and treatment of clinicians. Our study showed that the prevalence of high-risk HPV was 19.7% in women with CT infection or 21.8% in women with MG infection, which was not significantly more frequently detected than that in double-negative women. The study [33] reported that the genital HPV infection rate was 4.8% in CT-positive patients and 7.1% in CT-negative patients. There was no statistical difference between the two, which was consistent with our results. Previous study has reported a significant increase in the detection rate of HPV DNA in CT-positive patients [34], which was inconsistent with our results. Previous research suggested that the infection rate of MG increased significantly in women exposed to HPV infection [35]. Similarly, Xie *et al*. revealed that CT was a risk factor for high-risk HPV infection and MG was also a risk factor for cervical intraepithelial neoplasia [36]. Therefore, the relationship between MG or CT infection and high-risk HPV infection needs to be further explored, and the research population should be expanded, not limited to gynaecological patients. Moreover, there are diverse pathogens causing female genital tract infection, among which sexually transmitted pathogens include not only MG and CT, but also *Ureaplasma urealyticum*, *Mycoplasma hominis* and *Neisseria gonorrhoeae*. In future study, we will investigate the relationship between their separate infection, combined infection of two or more sexually transmitted pathogens and high-risk HPV infection.

The main limitation of this study may be that the previous treatment of each patient was not clear in our retrospective investigation, which interfered with the positive results of the target gene. Further prospective exploration is needed to exclude the impact of drugs on the experimental results. Besides, the subjects in our study were only from a single centre, so experimental results may have regional characteristics. In order to improve the infection of MG and CT in Chinese women, it is necessary to further carry out multi-centre studies.

## Conclusions

In conclusion, our study showed that the infection frequency of CT in gynaecological patients is significantly higher than that of MG, and a great percentage of patients infected by CT had symptoms of gynaecological diseases, while patients infected with MG were easily ignored because they were asymptomatic. Moreover, MG or CT infection will affect changes in the number of vaginal leukocytes and *Lactobacillus*, resulting in abnormal vaginal secretions, which may lead to vaginal diseases. However, there was no significant difference between MG or CT infection and high-risk HPV infection in women. Overall, our study explored the infection of MG and CT in women in Southwest China, which provided a basis for the clinical diagnosis and treatment of MG and CT infection.

## Data Availability

All data relevant to the study are included in the article. Not applicable.
